# Network Dismantling on Signed Network by Evolutionary Deep Reinforcement Learning

**DOI:** 10.3390/s24248026

**Published:** 2024-12-16

**Authors:** Yuxuan Ou, Fujing Xiong, Hairong Zhang, Huijia Li

**Affiliations:** School of Statistics and Data Science, Nankai University, Tianjin 300074, China; 2213927@mail.nankai.edu.cn (Y.O.); 2213033@mail.nankai.edu.cn (F.X.); 2212855@mail.nankai.edu.cn (H.Z.)

**Keywords:** signed network, network dismantling, evolutionary computation, deep learning, reinforcement learning

## Abstract

Network dismantling is an important question that has attracted much attention from many different research areas, including the disruption of criminal organizations, the maintenance of stability in sensor networks, and so on. However, almost all current algorithms focus on unsigned networks, and few studies explore the problem of signed network dismantling due to its complexity and lack of data. Importantly, there is a lack of an effective quality function to assess the performance of signed network dismantling, which seriously restricts its deeper applications. To address these questions, in this paper, we design a new objective function and further propose an effective algorithm named as DSEDR, which aims to search for the best dismantling strategy based on evolutionary deep reinforcement learning. Especially, since the evolutionary computation is able to solve global optimization and the deep reinforcement learning can speed up the network computation, we integrate it for the signed network dismantling efficiently. To verify the performance of DSEDR, we apply it to a series of representative artificial and real network data and compare the efficiency with some popular baseline methods. Based on the experimental results, DSEDR has superior performance to all other methods in both efficiency and interpretability.

## 1. Introduction

Although extensive research has been conducted in the fields of sensors and communication, ensuring the security of sensor networks remains an unresolved issue. This challenge is closely related to the technology of network dismantling. Network dismantling aims to determine a set of nodes (edges) to be removed from the network under specific constraints and various dismantling objective functions to minimize their overall network performance. It has gained significant prominence in the field of network science owing to its broad applications across various domains [1,2]. For example, by finding the most efficient set of nodes to dismantle a network, we can solve a number of problems such as dismantling criminal organizations [3,4], reducing the spread of rumors [5], controlling viruses [6], and maintaining the stability of sensor networks [7]. It is worth noting that network dismantling is a combinatorial optimization problem that has been proven to be NP-hard [8,9,10].

To find the optimal dismantling strategy, researchers have proposed some methods, which are described in detail in Section 2. While these methods have demonstrated efficiency in rapidly dismantling networks, they are primarily designed for addressing the issue of unsigned network dismantling. In fact, interactions between individuals in the real world may be given specific meanings [11,12]. For example, users may be friends or enemies in a social network, which necessitates the use of a signed network to represent the multi-type relationships between users [13]. In order to reflect the complexities of real-world networks accurately, the **signed network dismantling problem** has become an important and challenging area of research. Nevertheless, there are few studies that focus on signed network dismantling, and the main challenge is lack of efficient objective function and the corresponding algorithm, which consider both the sign and topological information.

Deep reinforcement learning [14] is a powerful technology that searches the optimal solution quickly through its reward mechanism and technique. However, it is easy to obtain the local optimal solution due to the local search of gradient descent [15]. In this paper, we propose a new framework for Dismantling on Signed Networks based on Evolutionary Deep Reinforcement Learning (**DSEDR**) to achieve fast search for global optimal solutions by combining the global search capability of evolutionary computation with the deep reinforcement learning. Specifically, we first propose a new objective function that considers both the connectivity of the network and the proportion of co-operative relationships (i.e., positive edges) in the signed network. By considering both features of sign information and network topology simultaneously, it transforms the signed network dismantling problem into a multi-objective optimization problem. To minimize this objective function, inspired by evolutionary computation and deep reinforcement learning methods [15], DSEDR integrates the advantages of both evolutionary computation and deep reinforcement learning to search for an optimal set of disassembled nodes to minimize the objective function and thus achieve the best dismantling result on a signed network. To obtain the network embedding, DSEDR initially employs an effective encoder to capture and learn the positive and negative topological characteristics. Then, it employs a Deep *Q*-Network (named as DQN) as a decoder to generate a dismantling strategy through a Markov decision process. In order to obtain the optimal weight parameters of the DQN, the algorithm optimizes the DQN by combining the global search capability of the evolutionary computation and the fast local search capability of the deep reinforcement learning. Finally, to verify the efficiency of DSEDR, we apply it on multi-type artificial and real network data sets. We compare the performance with eight popular baseline methods, and the experimental results demonstrate that DSEDR owns superior performance for signed networks dismantling among all the algorithms.

The subsequent sections are organized as follows: Section 2 presents an overview of related works, and Section 3 outlines the problem of dismantling on signed networks, and then establishes the objective function. Section 4 and Section 5 introduce the main framework and the detailed algorithm procedures of DSEDR. Section 6 demonstrates the experimental results, and finally, Section 7 concludes the whole work and provides future work.

## 2. Related Works

### 2.1. Network Dismantling Methods

Recently, network dismantling has received extensive attention from various disciplines, such as operations research, network science, and computer science. Many scientists focus on this important research area and have proposed some algorithms to explore this problem. Saeed et al. [16] introduced a network dismantling method based on network embedding, using geometric space representations to address the network dismantling problem. Sebastian et al. [17] developed a solution for the rapid identification of critical nodes across various network types, employing an iterative process that converts stochastic fault tracing into targeted attacks for effective dismantling. Additionally, Braunstein et al. [18] proposed the Min-Sum algorithm, a three-stage method that closely links the network dismantling problem to the de-ringing problem. Yan et al. [19] presented the HITTER framework, which transforms the hypernetwork dismantling to a sequential decision-making problem based on Deep Reinforcement Learning (DRL). Furthermore, Fan et al. [20] introduced FINDER, a Deep Reinforcement Learning-based approach designed to train intelligent agents applicable to a wide range of realistic networks. After all, network dismantling is still a new question and has not been fully studied. Moreover, there are few studies that focus on signed network dismantling, and the main challenge is lack of efficient objective functions and algorithms that consider both the sign information and network topology.

### 2.2. Evolutionary Deep Reinforcement Learning Algorithms

Reinforcement learning [21] is a learning mechanism that learns how to map from states to policies in order to maximize the reward obtained. The primary objective is to maximize cumulative rewards to identify a near-optimal solution for a given problem. Deep reinforcement learning algorithms integrate the perceptual capabilities of deep learning with the decision-making ability of reinforcement learning, optimizing neural network weights via backpropagation techniques to conduct effective searches in the action space of optimization problems. However, traditional deep reinforcement learning approaches predominantly rely on local search methods based on gradient descent, which can lead to local optimum [22]. To enhance the optimization performance of these algorithms, Kwon et al. [23] proposed the S2V-DQN algorithm, which integrates graph neural networks with deep *Q*-learning. This innovative approach allows agents to better comprehend the structural properties of graphs by embedding information about nodes and their neighbors into state representations.

The deep evolutionary algorithm represents an optimization method that combines evolutionary algorithms with deep learning to address complex non-convex optimization problems. This algorithm explores and optimizes solutions by applying evolutionary mechanisms to the strategy search process [24]. From a strategy search perspective, deep evolutionary algorithms can be classified into two main categories. The first category employs evolutionary algorithms to replace traditional strategy gradient-based optimization methods (e.g., gradient descent), facilitating more efficient strategy updates. This category includes popular approaches such as OpenAI-ES [25] and DRL-GA [26]. The second category treats policies as evolvable individuals, merging the population search characteristics of evolutionary algorithms with the gradient optimization capabilities of reinforcement learning, aiming to optimize populations of policies, such as EPG [23] and CERL [27].

## 3. Problem Formulation

### 3.1. Network Connectivity

Let G=(V,E) represent a given network, $V=\{V_{1},V_{2},\ldots ,V_{N}\}$ represent the set of nodes, and $E=[e_{1},e_{2},\ldots ,e_{w}]\subseteq V\times V$ represent the set of edges [28,29]. Here, N=|V| denotes the total number of nodes, while L=|E| indicates the total number of edges. To facilitate comprehension, the key symbols used in this paper are standardized and summarized in Table 1. There are multiple types of objective functions for traditional complex network dismantling problems, including the number of connected slices, the size of the giant connected component, the number of connected node pairs, etc. [30]. In this paper, we use the size of the giant connected component after network dismantling and select it as a part of the dismantling objectives due to its favorable properties [18]. It is important to note that this metric can be substituted with other conventional disintegration objectives, which depends on the problem being addressed.

Consider removing a subset of K nodes represented by the sequence of nodes $\{V_{1},V_{2},...,$$V_{K}\}$ in the network. The size of the giant connected component (GCC), denoted as σ, and the giant connected component size ratio, represented by $R_{\sigma }$, can be defined as follows:

(1) $$R_{\sigma }\left(\hat{V}\right)=\frac{\sigma (G-\hat{V})}{\sigma (G)},$$

where

(2) $$\sigma \left(G\right)=max\left\{\delta_{i},C_{i}\subset G\right\}.$$

Here, $C_{i}$ represents the *i*-th connected component and $\delta_{i}$ denotes the size of $C_{i}$ on the current graph G. The term $\sigma (G-\hat{V})$ refers to the size of GCC of the residual graph after sequentially removing nodes in the set $\hat{V}=\left\{V_{1},V_{2},\ldots ,V_{K}\right\}$ from G. Meanwhile, σ(G) represents the initial size of the GCC of G before any node removals.

### 3.2. Signed Networks

Signed networks, defined as a class of networked systems in which edges are labeled with “positive” and “negative” symbolic attributes, represent an effective tool for modeling social networks with emotional differences. The “positive and negative” symbols represent two opposing emotional attitudes. In particular, the positive edges are typically employed to characterize positive relationships, such as friendship, support, trust, and liking. These are indicated with a positive symbol, “+”. Negative edges are commonly used to represent negative relationships, such as enmity, opposition, distrust, and dislike. These are marked with a negative symbol, “−” [31].

The aforementioned meanings of positive and negative symbols can be applied to the signed network dismantling problem. In order to minimize the harmful impact on a malignant network, it is possible to decrease the proportion of cooperative/friendly relationships, i.e., positive edges, in the network as low as possible. Accordingly, the second optimization objective, which is denoted as $R_{pe}$, is defined as the percentage of positive edges in the signed network after the removal of nodes $\hat{V}$. This metric is calculated as follows:

(3) $$R_{pe}\left(\hat{V}\right)=\frac{k^{+}\left(G-\hat{V}\right)}{k(G-\hat{V})},$$

where $k^{+}\left(G-\hat{V}\right)$ is the number of positive edges of the residual graph after removing nodes in the set $\hat{V}=\left\{V_{1},V_{2},\cdots ,V_{K}\right\}$ sequentially from G, and k(G) is the total number of edges of the residual graph after removing nodes in the set $\hat{V}$.

### 3.3. The Objective Function

With the positive edge share $R_{pe}$ and the connectivity metric $R_{\sigma }$, the formula for the signed network dismantling objective function (denoted as Φ) is proposed as follows:

(4) $$\Phi \left(\hat{V}\right)=R_{\sigma }\left(\hat{V}\right)+R_{pe}\left(\hat{V}\right)\times \lambda ,$$

where $R_{\sigma }$ is the size of the GCC in Equation (1), $R_{pe}$ is the positive edge share in Equation (3), and $\hat{V}$ represents the set of nodes to be dismantled. λ denotes the importance of positive edge share in the signed network dismantling problem. It follows that, in general, the larger the value of λ, the greater the requirement for the reduction for positive edge share in the dismantled graph network. The value of λ is dependent upon the specific problem at hand and the graph to be dismantled. By reducing the objective function described above, it is possible to achieve a significant reduction in the connectivity of the network, while also taking into account the unique positive–negative edge relationship of the symbolic network, which reduces the cooperation ability between the nodes of the signed network. As a consequence, the objective function will facilitate the measuring of the degree of dismantling on the signed network.

The aforementioned definition of objective function conceptualizes the signed network dismantling problem as a multi-objective optimization problem. Our objective is to identify the optimal dismantling node set strategy, denoted by ${\hat{V}}_{op}$, that minimizes the objective function Φ, given the number of dismantling nodes K. The problem can be represented as follows:

(5) $${\hat{V}}_{op}=\underset{\hat{V}\subset G}{\mathrm{argmin}}\Phi \left(\hat{V}\right).$$

**Figure 1:**
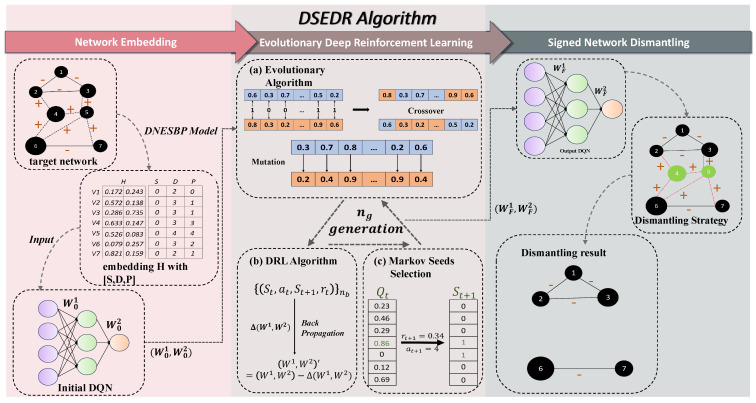
Flowchart of the DSEDR algorithm. Specifically, the algorithm consists of three parts. First, the ***Network Embedding*** procedure generates the embedding of the target network as the input of DQN, which transforms the problem into the optimization of DQN’s weight $\left(W^{1},W^{2}\right)$. Then ***Evolutionary Deep Reinforcement Learning*** is employed to find the best weight $\left(W_{F}^{1},W_{F}^{2}\right)$. Finally, ***Signed Network Dismantling*** is conducted by using DQN with the output weight.

In the next sections, we will propose a framework that provides a new strategy to remove the target set of nodes for optimizing the objective function, with the detailed process depicted in Figure 1.

## 4. Deep Reinforcement Learning

### 4.1. Network Embedding

In this paper, we reduce the network dimensionality reduction by extracting the low-dimensional features of a topological network through neural networks. This work uses the **Deep Network Embedding for Graph Representation Learning in Signed Networks** (DNESBP) algorithm [32] to perform network dimensionality reduction on signed networks, obtaining a *d*-dimensional feature representation vector $H={\left[h_{ij}\right]}_{n\times d}$, $h_{ij}\in \left[-1,1\right]$. The Deep *Q*-Network (named as DQN) can then input *H* to fully learn the positive and negative topological characteristics and structural balance properties of the signed network.

The DNESBP algorithm first uses a neural network with *ℓ* layers of neurons to construct an autoencoder to reduce the dimensionality of the signed network adjacency matrix *A* and obtain the representation learning vector, as follows:

(6) $$H^{\left(i\right)}=f\left(X^{\left(i\right)}{\left(W^{\left(i\right)}\right)}^{⊤}+B^{\left(i\right)}\right),\;i=1,\cdots ,\ell ,$$

where $X^{\left(0\right)}=A$ and $H^{\left(i\right)},i\neq 0$ is the vector obtained by the *i*-th dimensionality reduction. $H={\left[h_{ij}\right]}_{n\times d}$ denotes the vector of feature representations obtained by the final dimensionality reduction. The *f* function is the activation function used to generate positive and negative eigenvalues denoted as $f\left(x\right)=\left(\frac{e^{x}-e^{-x}}{e^{x}+e^{-x}}\right)$. $W^{\left(i\right)}$ is the network weight matrix in the *i*-th layer of the encoder, and $B^{\left(i\right)}$ is the bias vector of the network weights in the *i*-th layer of the encoder.

Next, the DNESBP algorithm reconstructs *H* by designing the decoder of the *l*-layer neural network and obtains the reconstruction matrix $\hat{A}$ as follows:

(7) $${\hat{X}}^{\left(i\right)}=f\left({\hat{H}}^{\left(i\right)}{\left({\hat{W}}^{\left(i\right)}\right)}^{⊤}+{\hat{B}}^{\left(i\right)}\right),\;i=1,\cdots ,\ell ,$$

where ${\hat{H}}^{\left(i\right)}=H^{\left(l\right)}$ and ${\hat{X}}^{\left(l\right)}=\hat{A}$, ${\hat{W}}^{\left(i\right)}$ is the network weight matrix of the decoder’s layer *i*, and ${\hat{B}}^{\left(i\right)}$ is the bias vector of the decoder’s layer *i* network weights. Further, the DNESBP algorithm supervises the dimensionality reduction process by computing the reconstruct. For the details of the loss function and the principle, please refer to [32].

### 4.2. Deep *Q*-Network

In our framework, the Deep *Q*-Network (DQN) [33] is implemented as a two-layer neural network specifically designed to approximate the optimal policy in reinforcement learning. The algorithm employs the dimensionality-reduced representation *H* as the input to DQN, which subsequently outputs a vector $Q=[q_{i}]$. Here, each $q_{i}$ corresponds to the *Q*-value associated with a specific node, and the node with the highest *Q*-value is selected for removal in the subsequent step.

**Figure 2:**
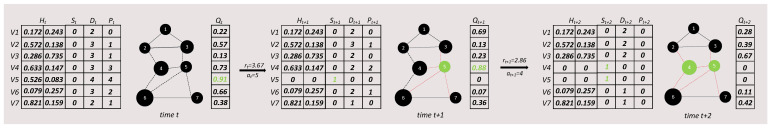
The flowchart of Markov seed selection, where $H_{t}$, $S_{t}$, $D_{t}$, and $P_{t}$ denote network embeddings, seed selection state vector, degree vector, and positive out-degree vector at time *t*, respectively. $Q_{t}$ is the *Q*-value vector; $r_{t}$ and $a_{t}$ are reward and action at *t*. Dashed lines represent positive edges, and solid lines represent negative edges.

It is important to note that the selection of a node for removal has an impact on an original network’s topology. Consequently, to reflect the changes in network dynamics resulting from the node selection, it is necessary to update the input vectors of DQN. Here, we propose a new approach to updating the inputs of DQN. The specific method is as follows: At each seed node selection, $S=\{s_{1},s_{2},\cdots ,s_{N}\}$ is used to denote the current seed node selection state, where $s_{i}=1$ and $s_{i}=0$ denote that node $V_{i}$ has been and has not been selected as a seed node, respectively. $D=\{d_{1},d_{2},\cdots ,d_{N}\}$ is the degree of each node of the current network, while $P=\{p_{1},p_{2},\cdots ,p_{N}\}$ is the positive out-degree of each node of the current network. Upon the selection of node $v_{i}$ at time t−1, the corresponding reduced feature H[i], degree $d_{i}$, and positive out-degree $p_{i}$ are set to zero, while $s_{i}$ is set to 1. The remaining elements in *D* and *P* will be updated by recomputing them based on the new network after removing node $v_{i}$. Then *H*, *S*, *D*, and *P* are spliced to form a new input vector. The detailed process can be seen in Figure 2.

DQN takes a (d+3)-dimensional vector as input and computes the *Q*-value from its network structure as follows:

(8) $$\begin{array}{cc} Q & =\mathrm{DQN}(W^{1},W^{2},\left[H_{t},S_{t},D_{t},P_{t}\right])\\ \; & =\mathrm{ReLU}\left(\left[H_{t},S_{t},D_{t},P_{t}\right]\cdot W^{1}\right)\cdot W^{2}. \end{array}$$

Here, $W^{1}$ represents the continuous weight matrix for the first layer of the neural network, while $W^{2}$ denotes the continuous weight vector of the second layer. The ReLU function is employed as the activation function to eliminate negative eigenvalues.

### 4.3. Markov Seed Selection

For the removed seed nodes, we employ a Markov decision process approach to optimize the iteration process and avoid selecting duplicated seed nodes [34]. Specifically, at time t−1, we first obtain the seed node sequence $S_{t-1}$. Then we update *H*, *S*, *D*, and *P* through the method mentioned in the DQN Module. At time *t*, $\left[H_{t},S_{t},D_{t},P_{t}\right]$ is input into the Deep *Q*-Network (DQN); meanwhile, we employ a greedy strategy to select the “node selection action” (Equations (9)–(11)) corresponding to the maximum *Q*-value, denoted as $a_{t}$. There are

(9) $$Q\left(S_{t},a\right)=\mathrm{DQN}\left(W^{1},W^{2},\left[H_{t},S_{t},D_{t},P_{t}\right]\right),$$

and

(10) $$Q\left(S_{t},a_{t}\right)=\max_{a}Q\left(\left[H_{t},S_{t},D_{t},P_{t}\right],a\right),$$

where

(11) $$a_{t}=\arg\max_{a}Q\left(S_{t},a\right).$$

Here, *a* represents the set of all possible actions that can occur when a removed seed node is selected, while $a_{t}$ denotes the specific action chosen at time *t*.

At time *t*, if a node $v_{i}$ has the highest *Q* value, the Markov decision process selects it as a seed node and makes $s_{i}=1$, and updates the sequence of seed nodes $S_{t}$, $D_{t}$$H_{t}$, and $P_{t}$. The current sequence of seed nodes $S_{t}$ is then converted to the set of seed nodes $V_{t}$ according to the following function O(S), and calculates the objective function Φ of the current signed network as follows:

(12) $$\begin{array}{cc} \Phi (V_{t}) & =\Phi (O\left(S_{t}\right))\\ s.t.\;O(S_{t}) & =\{V_{i}\in V:s_{i}=1\}. \end{array}$$

Next, the Markov decision process calculates the decision “reward” $r_{t}$, which is defined as the difference in objective function Φ before and after taking the action $a_{t}$ as follows:

(13) $$r_{t}=\Phi \left(V_{t}\right)-\Phi \left(V_{t+1}\right).$$

By using the DQN Module and Markov Decision Module, DSEDR transforms the discrete combination optimization problem of selecting K seed nodes in a signed network into the continuous parameter optimization problem of DQN as follows:

(14) $$\begin{array}{cc} \min\; & \Phi \left(S\right)=\Phi \left(W^{1},W^{2}\right)\\ s.t.\; & W^{1},W^{2}\in DQN. \end{array}$$

The MSS Algorithm can be seen in Algorithm 1. In the next section, we propose a new evolutionary deep reinforcement learning algorithm DSEDR to resolve this parameter optimization problem of DQN.
**Algorithm 1** MSS (Markov seed selection) Algorithm**Input:** Parameters $\left(W^{1},W^{2}\right)$, size of seed set K and target network G.**Output:** Seed set $\hat{V}$, $\Phi (\hat{V})$ and a set of Markov decision processes ${\left\{\left(S_{t},a_{t},r_{t},S_{t+1}\right)\right\}}_{K}$.   1:Initialization: set t←1 and $S_{t}\leftarrow$ [ ];   2:Computing degree $d_{i}$ and positive out-degree $p_{i}$ of each node $v_{i}$ in G, then set $D_{t}\leftarrow {\left[d_{i}\right]}_{N}$ and $P_{t}\leftarrow {\left[p_{i}\right]}_{N}$;   3:Set DQN $\leftarrow (W^{1},W^{2})$;   4:**while **t≤K** do**   5:   Compute $Q(S_{t},a)$ based on Equation (9);   6:   Compute $a_{t}$ based on Equation (11), which selects the node with the highest *Q*-value and then obtain $S_{t+1}$;   7:   Update $H_{t}$, $D_{t}$, and $P_{t}$ according to $a_{t}$ and obtain $H_{t+1}$, $D_{t+1}$, and $P_{t+1}$;   8:   Compute $r_{t}=\Phi \left(V_{t}\right)-\Phi \left(V_{t+1}\right)$;   9:   t←t+1; 10:**end while** 11:$B\leftarrow {\left\{\left(S_{t},a_{t},r_{t},S_{t+1}\right)\right\}}_{K}$, $\hat{V}\leftarrow V_{K}$; 12:return $\hat{V},\Phi \left(\hat{V}\right)$;

## 5. The DSEDR Algorithm

In this section, we introduce the detailed procedures of the DSEDR algorithm designed to search for the optimal solution for the signed network dismantling problem in Equation (5). We aim to integrate the advantages of evolutionary computation and deep reinforcement learning to facilitate the optimization process.

### 5.1. Evolution of DQN Populations

#### 5.1.1. Solution Representation and Evaluation

In DSEDR, all individuals in a population $P={\left\{P_{i}\right\}}_{n_{p}}$ evolve at the same time, where each solution $P_{i}$ represents a DQN. It can be represented as follows:

(15) $$P_{i}=\left(W_{i}^{1},W_{i}^{2}\right),W_{i}^{1}\in {\left[-1,1\right]}_{(d+3)\cdot l},W_{i}^{2}\in {\left[-1,1\right]}_{l\cdot 1},$$

where the weight parameters $W^{1}$ and $W^{2}$ correspond to the first and second layers of DQN, respectively. The number of neural nodes in the first and second layers is given by d+3 and *l*, respectively.

For each solution, DSEDR combines the network embedding, DQN, and Markov seed selection modules described above with the objective in Equation (5) to generate two outputs. One output is the set of seed nodes (denoted by ${\hat{V}}^{i}$) and $\Phi^{i}$, which are used in the evolutionary step to evaluate the fitness of the solution (denoted by $P_{i}$). The other output consists of a set of Markov decision processes, i.e., sequences of states, actions, rewards, and subsequent states, which are used to accelerate the DQN optimization in the deep reinforcement learning step.

#### 5.1.2. Initialization Operations

DSEDR initially randomizes the weight sequence population of the DQN network in order to obtain the initial solution set for the DQN population. This random initialization operation ensures the diversity of the initial DQN population solutions. The specific procedure for this is outlined in Algorithm 2. In particular, a value is randomly generated for each weight parameter of DQN, ranging from −1 to 1, which serves as the initial value.
**Algorithm 2** Initialization Algorithm**Input:** Initial population size: $n_{I}$.**Output:** Initial solutions P. 1:Set $P={\left\{P_{i}\left(0\right)\right\}}_{n_{I}},P_{i}\left(0\right)=\left(W_{i}^{1},W_{i}^{2}\right),i=1,2,3,\cdots ,n_{I}$; 2:**for **$i=1,2,3,\cdots ,n_{I}$** do** 3:   **for** each element in $W_{i}^{1},W_{i}^{2}$ **do** 4:     Randomly generate a value in the range of −1 to 1; 5:   **end for** 6:**end for**

#### 5.1.3. Evolutionary Operations

Following the initial acquisition of the DQN population, the DSEDR algorithm initiates an iterative process of evolution, including crossover, mutation, and selection operations on DQN. These are conducted with the objective function Φ as the fitness value. These operations are illustrated in the subsequent description.

The crossover operation focuses on the parent solution P(q), which first orders the population set based on fitness values, and then generates new offspring using two operations, solution pairing and single-point crossover. The solution pairing divides the parent solution P(q) into the set with “good genes” $\left\{P_{i}^{b}\left(q\right)\right\}$ and the set with “average genes” $\left\{P_{j}^{w}\left(q\right)\right\}$. For each pair of solutions $\left\{P_{i}^{b}\left(q\right),P_{j}^{w}\left(q\right)\right\}$, the single-point crossover first generates a random bit-value of either 0 or 1 for each gene position, and then crosses over the gene values with a bit-value of 1 in the two solutions to generate the offspring $\left\{P_{i}{\left(q\right)}^{C}\right\}$ and $\left\{P_{j}{\left(q\right)}^{C}\right\}$, and we write down all the offspring generated by the crossover operation as follows:

(16) $$\left\{P{\left(q\right)}^{C}\right\}=\left\{P_{i}{\left(q\right)}^{C}\right\}\cup \left\{P_{j}{\left(q\right)}^{C}\right\}.$$

For mutation operation, a random mutation is performed by the following *N* function for each gene $y_{i}$ in the population to be mutated, which enhances the diversity of the population’s gene sequences and results in a mutated progeny $\left\{P{\left(q\right)}^{M}\right\}$, which is presented in the following equation:

$$y_{i}=N\left(-1,1\right),$$

where the *N* function is a normal random function used to generate values from −1 to 1, and obeys the probability density function $\frac{1}{\sqrt{2\pi }}\cdot e^{-y^{2}/2}$.

The selection operation is based on a greedy strategy, whereby the population $P{\left(q\right)}_{EA}$ with the highest fitness value Φ(V) is selected from $P{\left(q\right)}^{C}\cup P{\left(q\right)}^{M}$. Subsequently, the population $P{\left(q\right)}_{EA}$, which comprises “superior genes”, is chosen as the population to be evolved in the next generation. Refer to Algorithm 3 for details of the evolution procedure.
**Algorithm 3** Evolution Algorithm**Input:** Population size $n_{p}$, parent solutions P(q), crossover probability $p_{crossover}$, and mutation probability $p_{mutation}$.**Output:** Offspring solutions: $P{\left(q\right)}_{EA}$.   1:Randomly divide P(q) into two populations: $P{\left(q\right)}_{C}$ and $P{\left(q\right)}_{M}$;   2:$\hat{V},\Phi \left(\hat{V}\right)\leftarrow \mathrm{MSS}\left(P{\left(q\right)}_{C},K,H\right)$;   3:Sort the solutions $P{\left(q\right)}_{c}$ in an ascending order based on their $\Phi (\hat{V})$;   4:Compose $\left\{P_{i}^{b}\left(q\right),P_{j}^{w}\left(q\right)\right\}$ of the first 50% of the populations $\left\{P_{i}^{b}\left(q\right)\right\}$ and the last 50% of the populations $\left\{P_{j}^{w}\left(q\right)\right\}$;   5:**for** each pair of solutions $\left(P_{i}^{b}\left(q\right),P_{j}^{w}\left(q\right)\right)$ **do**   6:   **if** A randomly generated value $p_{1}\leq p_{crossover}$ **then**   7:     Each pair of genes $\left(x_{i},x_{j}\right)$ in $P_{i}^{b}\left(q\right)$ and $P_{j}^{w}\left(q\right)$ is randomly assigned a value of 0 or 1, thus constituting the sequence *M*;   8:     **for** each $\left(x_{i},x_{j}\right)$ in $\left\{P_{i}^{b}\left(q\right),P_{j}^{w}\left(q\right)\right\}$ **do**   9:        **if** $\left(x_{i},x_{j}\right)$ corresponds to a value of 1 in the sequence *M* **then** 10:          Replace the values of $x_{i}$ and $x_{j}$ to obtain a new $P_{i}{\left(q\right)}^{C}$ and $P_{j}{\left(q\right)}^{C}$; 11:        **end if** 12:     **end for** 13:   **end if** 14:**end for** 15:Set $\left\{P{\left(q\right)}^{C}\right\}=\left\{P_{i}{\left(q\right)}^{C}\right\}\cup \left\{P_{j}{\left(q\right)}^{C}\right\}$; 16:**for** each solution $P_{i}{\left(q\right)}_{M}$ in $P{\left(q\right)}_{M}$ **do** 17:   **for** each weight parameter y in $P_{i}{\left(q\right)}_{M}$ **do** 18:     **if** A randomly generated value $p_{2}\leq p_{mutation}$ **then** 19:        Update y value to a value N(−1,1),get $P_{i}{\left(q\right)}^{M}$; 20:     **end if** 21:   **end for** 22:**end for** 23:$\hat{V},\Phi \left(\hat{V}\right)\leftarrow \mathrm{MSS}\left(P{\left(q\right)}^{C}\cup P{\left(q\right)}^{M},K,H\right)$; 24:The $n_{p}$ populations from $P{\left(q\right)}^{C}\cup P{\left(q\right)}^{M}$ with the highest $\Phi (\hat{V})$ are selected as $P{\left(q\right)}_{EA}$;

### 5.2. Reinforcement Learning Operation

In large-scale networks, the numerous weight sequences associated with DQN make it difficult to find an optimal solution within limited iterations, resulting in slow convergence speed. To address this problem, DSEDR introduces an *n*-step *Q*-learning technique based on deep reinforcement learning concepts to accelerate DQN training and evolution.

DSEDR stores the historical quaternion data set, including the quadruplet ${\left\{\left(S_{t},a_{t},r_{t},S_{t+1}\right)\right\}}_{n_{g}}$ in the cache pool B, throughout the iterative seed node selection process conducted in accordance with the Markov decision process. Following $n_{g}$ iterations of training, the DSEDR algorithm samples quaternion data in batches from the cache pool based on the $n_{b}$-step *Q*-learning technique. These data are then used to compute the loss function $L(W^{1},W^{2})$ of DQN with weight parameters $\left(W^{1},W^{2}\right)$, which evaluates the expected difference between predicted Q values and target Q values. The loss function can be computed as follows:

(17) $$L\left(W^{1},W^{2}\right)=E_{C\in B}\left[{\left(\underset{\mathrm{predicted}\;Q\;\mathrm{values}}{\underset{︸}{\sum_{i=0}^{n_{b}-1}r_{t+i}+\gamma \max Q\left(S_{t+n_{b}},a^{*};W^{1},W^{2}\right)}}-\underset{\mathrm{target}\;Q\;\mathrm{values}}{\underset{︸}{Q(S_{t},a_{t};W^{1},W^{2})}}\right)}^{2}\right],$$

where γ denotes the hyperparameter used to determine the importance of the reward $r_{t+i}$ and *E* denotes the expectation function. B denotes the cache pool used to store the quaternion data, and $C={\left\{\left(S_{t},a_{t},r_{t},S_{t+1}\right)\right\}}_{n_{b}}$ denotes that each batch is taken out from the cache pool B for updating the quaternion data of DQN with a batch size of $n_{b}$. Based on the above objective loss function $L(W^{1},W^{2})$, the DSEDR algorithm can further compute the gradient of the loss function $\Delta (W^{1},W^{2})$, which can be computed as follows:

(18) $$\begin{array}{cc} \Delta (W^{1},W^{2})= & \alpha \left[\sum_{i=0}^{n_{b}-1}r_{t+i}+\gamma \max Q\left(S_{t+n_{b}},a^{*};W^{1},W^{2}\right)-Q\left(S_{t},a_{t};W^{1},W^{2}\right)\right]\\ \; & \cdot \nabla_{\left(W^{1},W^{2}\right)}Q\left(S_{t},a_{t};W^{1},W^{2}\right), \end{array}$$

where α denotes the learning rate when updating DQN in reverse, and $\nabla_{\left(W^{1},W^{2}\right)}$ denotes the deviation to $\left(W^{1},W^{2}\right)$.

Then DSEDR adopt the following stochastic gradient descent algorithm to inversely update the network weights of the DQN $\left(W^{1},W^{2}\right)$:

(19) $${\left(W^{1},W^{2}\right)}^{\prime }=\left(W^{1},W^{2}\right)-\Delta \left(W^{1},W^{2}\right).$$

Following the aforementioned operation, the convergence of the network weights of DQN can be accelerated towards superior weights. Refer to Algorithm 4 for details of the DRL procedure.
**Algorithm 4** DRL (deep reinforcement learning) Algorithm**Input:** Cache pool B, batch size $n_{b}$, population $P{\left(q\right)}_{EA}$.**Output:** Population $P{\left(q\right)}_{DRL}$. 1:Randomly retrieve $C={\left\{\left(S_{t},a_{t},r_{t},S_{t+1}\right)\right\}}_{n_{b}}$ of size $n_{b}$ from the cache pool B; 2:Calculating $L(W^{1},W^{2})$ by Equation (17); 3:Calculating $\Delta (W^{1},W^{2})$ of $L(W^{1},W^{2})$ by Equation (18); 4:Based on $\Delta (W^{1},W^{2})$, reverse update $\left(W^{1},W^{2}\right)$ corresponding to the most favorable population $P{\left(q\right)}_{EA}$, thus obtaining a new population $P{\left(q\right)}_{DRL}$;

### 5.3. DSEDR Algorithm

DSEDR combines evolutionary computation with deep reinforcement learning to enhance the evolution of DQNs. The evolutionary computation concurrently evolves a population of individuals, where each individual embodies a DQN, and ultimately derives a solution to the signed network dismantling problem through the previously outlined seed selection strategy. In contrast, deep reinforcement learning leverages the combined information and network-specific insights of the DQNs to speed up their evolutionary process.

More specifically, in each iteration, the DSEDR algorithm employs a two-step approach to optimize the DQN network weight parameters. First, it searches globally through the crossover and mutation of the genetic algorithm. Second, it searches locally through the stochastic gradient descent algorithm of reinforcement learning. Additionally, it introduces the *n*-step *Q*-learning technique, which makes use of the quaternions stored in the cache pool for the back propagation to update the DQN network weights. The network weights of DQNs are updated in order to accelerate the training convergence and evolution of DQNs. Furthermore, DQN networks are evaluated and screened by the use of a Markov decision process and the aforementioned objective function Φ in Equation (4). The part of the population with the highest fitness value is selected, while the other part of the population is randomly selected for the next iteration. This approach ensures the evolution of the population and the diversity of stochasticity, which can be achieved by the use of the greedy strategy.

### 5.4. Complexity Analysis

As previously stated, DSEDR (Algorithm 5) includes four different sub-algorithms: MSS (Algorithm 1), Initialization (Algorithm 2), Evolution (Algorithm 3), and DRL (Algorithm 4). The computational complexity of each sub-algorithm will be calculated separately and subsequently combined to derive the overall algorithmic complexity of DSEDR.

- **MSS**: The time complexity of MSS can be computed as $O(K\times \left(d\times l+\bar{K}\times \bar{K}\right))$, where K is the number of nodes to be removed, $\bar{K}$ is the average connectivity of nodes in the target network, *d* and *l* denote the number of neurons in the first and the second layers of the DQN, respectively.
- **Initialization**: The time complexity of Initialization can be computed as $O(n_{I}\times d\times l)$, where $n_{I}$ is the initial population size.
- **EA**: The time complexity of EA can be computed as $O(n_{I}\times K\times \left(d\times l+\bar{K}\times \bar{K}\right))$.
- **DRL**: The time complexity of DRL can be computed as $O(n_{b}\times \left(d\times l+\bar{K}\times \bar{K}\right))$, where $n_{b}$ is the batch size in the DRL Algorithm.

**Algorithm 5** DSEDR Algorithm
**Input:** Initial population size: $n_{I}$, maximum number of iterations $n_{g}$, population size $n_{p}$, parent solutions P(q), crossover probability $p_{crossover}$, and mutation probability $p_{mutation}$. 1:${\left\{P_{i}\left(0\right)\right\}}_{n_{I}}\leftarrow \mathrm{Initialisation}\left(\mathrm{Algorithm}\;2\right)\;\left(n_{I}\right)$; 2:**for** q = 0 to $n_{g}-1$ **do** 3:   $P{\left(q\right)}_{EA}\leftarrow \mathrm{Evolution}\left(\mathrm{Algorithm}\;3\right)\;\left(n_{p},P\left(q\right),p_{crossover},p_{mutation}\right)$; 4:   $P{\left(q\right)}_{DRL}\leftarrow \mathrm{DRL}\left(\mathrm{Algorithm}\;4\right)\;\left(P{\left(q\right)}_{EA}\right)$; 5:   $V,\Phi \left(V\right)\leftarrow \mathrm{MSS}\left(\mathrm{Algorithm}\;1\right)\;(P\left(q\right)\cup P{\left(q\right)}_{EA}\cup P{\left(q\right)}_{DRL},K,H)$ 6:   Select population with the highest Φ(V) and size of $n_{p}/2$ from $P\left(q\right)\cup P{\left(q\right)}_{EA}\cup P{\left(q\right)}_{DRL}$ as $P{\left(q\right)}_{Opt}$; 7:   Randomly select population with the size of $n_{p}/2$ from $P\left(q\right)\cup P{\left(q\right)}_{EA}\cup P{\left(q\right)}_{DRL}-P{\left(q\right)}_{Opt}$ as $P{\left(q\right)}_{Random}$; 8:   $P\left(q+1\right)\leftarrow P{\left(q\right)}_{Random}\cup P{\left(q\right)}_{Opt}$; 9:
**end for**



By summing up all the complexities of the four sub-algorithms, for a total of $n_{g}$ iterations, the total time complexity of DSEDR can be computed as follows:

(20) $$O(n_{g}\times \left(n_{I}\times K+n_{b}\right)\times \left(d\times l+\bar{K}\times \bar{K}\right)).$$

It is worth noting that in sparse networks, since the number of links is nearly linear with the number of nodes, we can further reduce this complexity to $O(n_{g}\times \left(n_{I}\times K+n_{b}\right)\times \left(d\times l+\theta \right))$, where θ is a constant.

## 6. Experiments

In this section, we compare the performance of DSEDR with those of various baseline methods on multiple-type network datasets to verify its efficiency. Specifically, we first provide a brief description of the experimental settings and then show the experiment results on artificial networks and a real-world network, respectively. All experiments were conducted on a server running Linux, equipped with a 16-core AMD EPYC 9354 CPU, 60.1 GB of memory, an NVIDIA RTX 4090 GPU with 25.2 GB of graphics memory, and a storage disk with 751.6 GB capacity.

### 6.1. Experimental Settings

#### 6.1.1. Baseline Algorithm

Because there are few methods that have been proposed for signed network dismantling, the selection of the baselines is primarily based on classical centrality methods and excellent conventional algorithms in complex network dismantling. In this paper, we select 10 classical centrality metrics and 2 conventional algorithms, MinSum [18] and FINDER [20], as baselines. These classical centrality metrics include metrics that are not related to edge symbols, such as Degree [35], Betweenness [36], K-shell [37], and Closeness [38], as well as metrics that take edge symbols into account, such as Positive degree (P-DEG), Negative degree (N-DEG), Net degree (Net-DEG), Ratio degree (Ratio-DEG), Prestige [39], and PageRank [40]. A brief description of these baselines is provided below.

- **Degree:** The degree of a node, i.e., the number of neighboring nodes directly connected to the node [35].
- **Betweenness:** Betweenness Centrality (BC) reflects how often a node appears on the shortest paths between pairs of other nodes. The BC of a node is defined as follows:
  (21) $$BC\left(i\right)=\sum_{i\neq s,j\neq t,s\neq t}\frac{g_{st}^{i}}{g_{st}},$$
  where $g_{st}$ is the number of all shortest paths from node $v_{s}$ to $v_{t}$, and $g_{st}^{i}$ is the number of shortest paths passing through $v_{i}$ among the $g_{st}$ shortest paths from node $v_{s}$ to $v_{t}$ [36].
- **K-shell:** K-shell centrality categorizes nodes based on their degrees to assess their importance in a network. Assuming there are no isolated nodes in the network, we eliminate nodes with one connection until no such nodes remain and assign them to the 1-shell. Similarly, we recursively eliminate nodes with degree 2 to form the 2-shell. This process ends when all nodes have been assigned to one of the shells [37].
- **Closeness:** Closeness Centrality reflects the distance between a node and all other nodes in the network and measures the average shortest path length from the node to all other nodes. A higher closeness value indicates a more central position within the network. It can be computed as follows:
  (22) $$d_{i}=\frac{N-1}{\sum_{i\neq j}d_{ij}},$$
  where $d_{ij}$ is the length of the shortest path between node $v_{i}$ and node $v_{j}$ [38].
- **Positive degree (P-DEG):** The number of positive edges connected to the *i*-th node, denoted as $k_{i}^{+}$.
- **Negative degree (N-DEG):** The number of negative edges connected to the *i*-th node, denoted as $k_{i}^{-}$.
- **Net degree (Net-DEG):** This metric represents the difference between P-DEG and N-DEG:
  (23) $$k_{i}^{+}-k_{i}^{-}.$$
- **Ratio degree (Ratio-DEG):** It indicates the proportion of positive edges connected to node $v_{i}$ relative to its total edges in the network, as follows:
  (24) $$\frac{k_{i}^{+}}{k_{i}^{+}+k_{i}^{-}}.$$
- **Prestige:** Prestige is determined by both the positive and negative incoming links to a node [39]. The prestige of node *i* ($Pr_{i}$) is calculated as follows:
  (25) $$Pr_{i}=\frac{k_{i}^{+}-k_{i}^{-}}{k_{i}^{+}+k_{i}^{-}}$$
- **PageRank:** PageRank, which was inspired by Larry Page of Google, is among the most prevalent ranking algorithms in use today [40]. We can represent the PageRank score of node *i* as PR(i). This rank value is computed (in an iterative manner) as follows:
  (26) $$PR_{i}\left(t+1\right)=\alpha \sum_{j\in IN_{i}}\frac{PR_{j}\left(t\right)}{|OUT_{j}|}+\left(1-\alpha \right)\frac{1}{N}$$
  where α is a forgetting factor. N is the number of nodes in the network. $IN_{i}$ represents the set of nodes that have edges pointing to node *i*, and $|OUT_{j}|$ represents the number of outgoing links from node *j*.
- **MinSum and FINDER:** Two outstanding algorithms in complex network dismantling. For more information, please refer to [18,20].

#### 6.1.2. Parameter Setting

Next, we present the specific parameter setting regarding the evolutionary algorithm and the DQN network in the experiments, which contain the number of iterations, population size, training batch size, etc., as detailed in Table 2.

Among these parameters, we give the results of the influence of crossover and mutation probabilities on the DSEDR algorithm in the following experiments to demonstrate that DSEDR maintains a stable dismantling efficiency under different evolutionary probability settings.

### 6.2. Artificial Network

In our experiments with artificial networks, we choose the famous LFR network [41] and the BA network [42].

The LFR network is a model for generating complex networks with an intrinsic community structure. In LFR networks, the mixing parameter μ controls the proportion of cross-community edges in the total edges of nodes. A smaller mixing parameter indicates a clearer community structure and more connected nodes within each community. In our experiments, we set the power law exponent of the node degree distribution of networks β to 3 and the power law exponent of the community size distribution γ to 1.5, and construct 5 medium-sized LFR networks with 1000 nodes by setting 5 different mixing parameters μ.

The BA network is a model for generating scale-free networks, characterized by a power-law degree distribution. In this model, a few nodes (high-degree nodes) have a large number of connections, while the majority of nodes have only a few connections. In the BA model, the parameter ω represents the number of connections each new node makes to existing nodes. A larger value for this parameter increases the network’s overall connectivity, making the power-law feature more significant. In our experiments, we construct 5 medium-sized BA networks with 1000 nodes by setting 5 different parameters ω.

**Figure 3:**
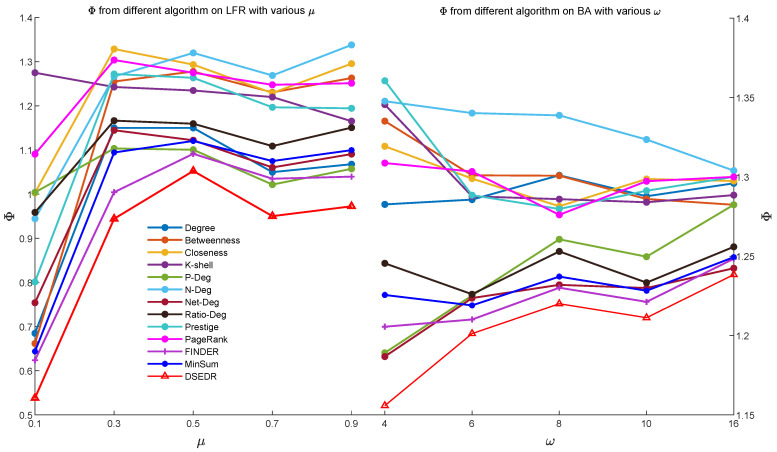
Efficiency comparison on artificial networks. Among 13 algorithms, DSEDR has the best performance in the LFR network under 5 different mixing parameters μ and in the BA network under 5 different parameters ω.

**Figure 4:**
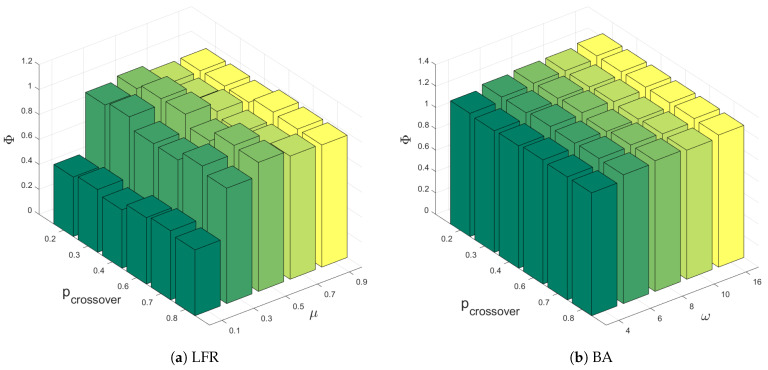
Influence of $p_{crossover}$ on artificial networks. Specifically, (**a**) is the result of a different $p_{crossover}$ on LFR with a different μ, and (**b**) shows the result of a different $p_{crossover}$ on BA with a different ω.

Due to the inherent randomness in our algorithm, we conduct 20 independent experiments using DSEDR to dismantle 10% nodes on each network. Figure 3 presents the results of 20 experiments in a box plot and compares the efficiency of DSEDR with 8 baselines. Figure 4 illustrates the impact of $p_{crossover}$ on the performance of DSEDR across various artificial networks. For each value of $p_{crossover}$, we conduct 20 experiments and then plot the mean value of the objective function Φ in a bar plot for comparison. The results show that our algorithm consistently outperforms the 12 baselines on 10 artificial networks under different μ and ω settings, and remains stable under different $p_{crossover}$ settings.

**Figure 5:**
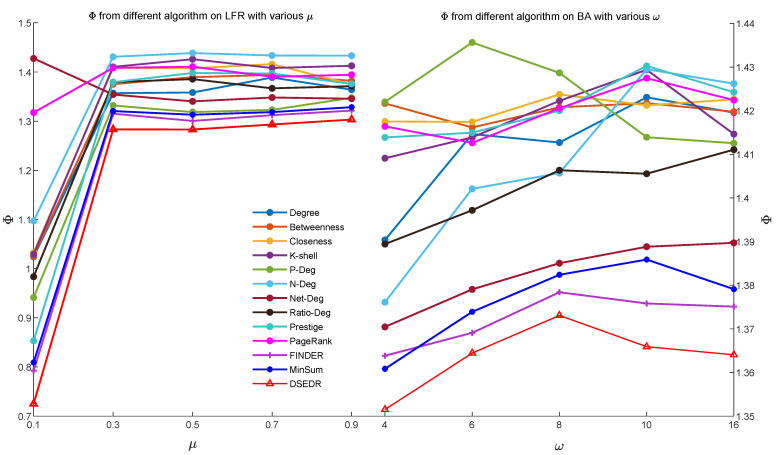
Efficiency comparison on 10 different networks with 10,000 nodes after dismantling 10% nodes.

To verify that DSEDR still maintains its high efficiency on large-scale networks, we also conducted the same experiments on the LFR and BA networks with 10,000 nodes. The results are shown in Figure 5, which demonstrate that even in large-scale networks, DSEDR still exhibits excellent efficiency.

### 6.3. Real-World Network

For the real network dismantling experiment, we choose the war network as the experimental network. The war network is a network data set of military relationships extracted from the Military Warfare Project [43], which reflects the alliances and antagonisms constituted by 166 countries between 2000 and 2010. The positive edge represents an alliance relationship between two countries, while the negative edge represents an antagonistic relationship between two countries. The detailed data information of this network is shown in Table 3. In this part, we present the dismantling efficiency comparison of the DSEDR algorithm with 8 baselines on the war network and the influence of parameter $p_{crossover}$ on DSEDR’s dismantling performance. Further more, the visualization of the dismantling procedure and the analysis of real-world meaning of the dismantling are given as well.

#### 6.3.1. Efficiency and Parameter Analysis

**Figure 6:**
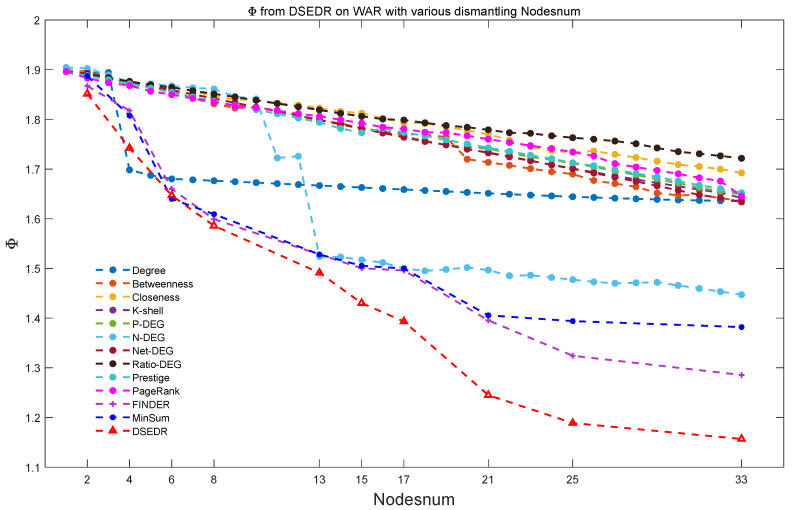
Efficiency comparison on the war network. Among 9 algorithms, DSEDR has excellent performance compared with all 12 baselines for 10 different numbers of removed nodes.

Figure 6 shows the efficiency comparison of DSEDR and 8 baselines. In the experiment, we select 10 various numbers of dismantled nodes. Considering the randomness in the initialization part of the algorithm, we conduct 20 experiments for each number of dismantled nodes, respectively. Subsequently, we present the results as a box plot for a comparison with the 12 baselines. It is evident that the DSEDR algorithm is much more effective in optimizing the objective function Φ than the baselines. In particular, when the number of removed nodes reaches 20% of the total points (i.e., 33 nodes), the DSEDR algorithm exhibits an apparent advantage over the baselines.

**Figure 7:**
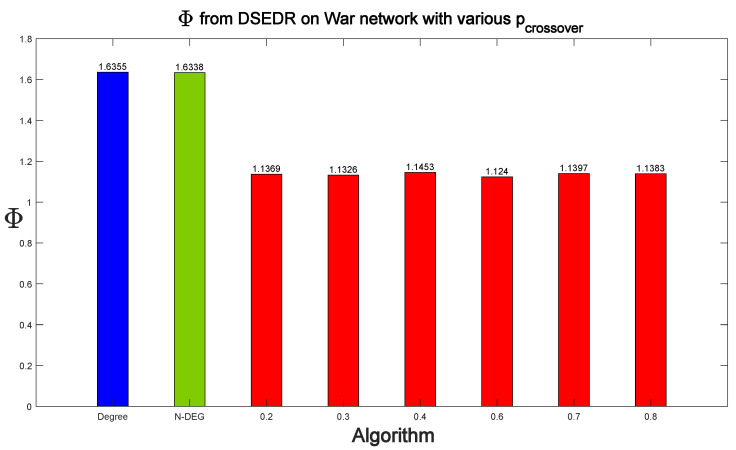
Influence of $p_{crossover}$ on the war network, where red bars denote the efficiency of DSEDR with different $p_{crossover}$. Here, we select the top 2 baselines, Degree and Net-DEG, to be compared.

Figure 7 shows the influence of the parameter $p_{crossover}$ on DSEDR’s performance when 33 nodes are dismantled. In order to compare DSEDR with 12 baselines more concisely, we choose only the two baselines with the best dismantling result, Degree and N-DEG, for comparison here. We conduct 20 experiments with DSEDR at each different $p_{crossover}$, and the mean values of the obtained Φ are plotted as a bar plot to compare with the 2 baselines. The performance of the DSEDR algorithm is relatively stable under different $p_{crossover}$ and remains significantly more efficient than the baselines under different parameter settings according to the figure.

#### 6.3.2. Visualization and Real Meaning Analysis

**Figure 8:**
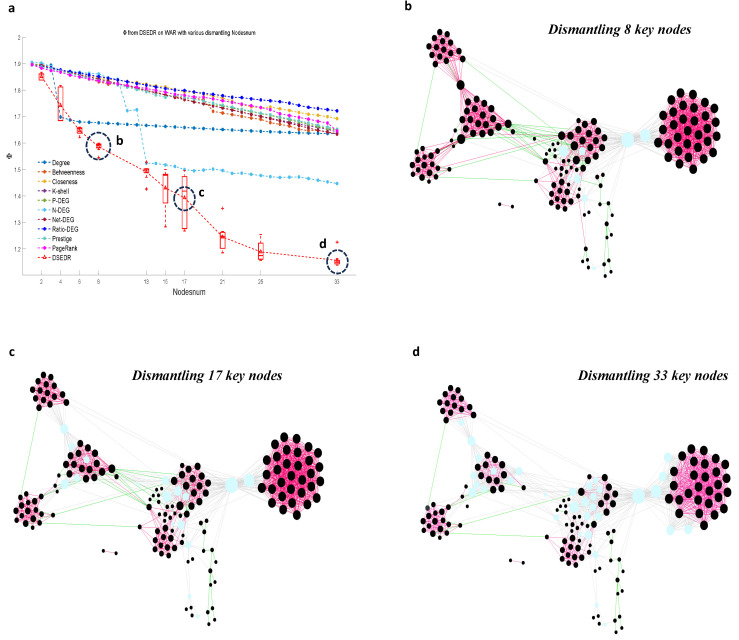
The process of dismantling the war network using DSEDR. DSEDR seeks to design a node removal sequence to minimize the objective function Φ in Equation (4). (**a**) illustrates the objective function curve on the war network with the horizontal axis being the number of dismantled nodes and the vertical axis being the Φ of the residual graph after removing these nodes. (**b**–**d**) show the snapshots after removing 8 (**b**), 17 (**c**), and 33 (**d**) key nodes(cyan) determined by DSEDR at the different time points marked in the objective function curve of DSEDR in (**a**), respectively. The red line denotes the positive edge, and the green line denotes the negative edge.

To further demonstrate the effect and application value of the DSEDR algorithm in signed network dismantling, this part presents the visualization of the war network dismantling process using the DSEDR algorithm, i.e., Figure 8. The figure comprises three snapshots, (b), (c), and (d), each displaying the war network after dismantling different numbers of nodes during the process (a).

As we can see from Figure 8d, DSEDR dismantles the complex coalition-versus relationship among 166 countries by removing 33 of the nodes (which can be interpreted as isolating the diplomatic relations of these countries) and breaks them down into clearly structured components of coalition nodes, antagonistic nodes, and a number of independent nodes. Unlike traditional complex network dismantling, DSEDR does not only seek to reduce the size of the giant connectivity component in signed network dismantling. When the structure of the node clusters is relatively clear, DSEDR further seeks to increase the proportion of negative edges in the network so as to reduce the stability of the alliance and confrontation network formed by these 166 countries, which is able to give an opinion on the maintenance of the international peace.

## 7. Conclusions

In this paper, we propose a new framework named DSEDR to explore the signed network dismantling problem that integrates the advantages of both evolutionary computation and deep reinforcement learning. First, we propose a new objective function that integrates features of sign information and network topology. Then, we transform the objective function minimization to a continuous parameter optimization of a deep *Q*-learning network. To obtain the optimal parameter, DSEDR utilizes evolutionary computation by considering different DQN parameters as different population individuals, and searches for the optimal DQN parameters by combining the global search capability in evolutionary algorithms with the fast local search capability in deep reinforcement learning. Finally, to verify the efficiency of DSEDR, we apply it to multiple-type artificial and real network data sets. We compare the performance with 12 popular baseline methods, and the experimental results demonstrate that DSEDR owns superior performance for signed networks dismantling among all the algorithms. Due to its demonstrated superiority in signed network dismantling problems, DSEDR has great application value, such as disrupting rumor propagation networks, finding critical components in sensor networks, and maintaining sensor network stability.

In future research, we will further extend the application of networks to a more large-scale network with millions of nodes. Moreover, with the development of high-order network studies, we can apply DSEDR on more complex topologies, such as directed networks and temporal networks. Meanwhile, we can evolve the parameter encoder together with the parameters of DQN, which can further improve the performance of our framework.

## Figures and Tables

**Table 1:** Summary of notation.

Notation	Instruction
G	Target network
V	Set of nodes
E	Set of edges
N	Number of nodes
L	Number of edges
σ	Size of giant connected component
$C_{i}$	The *i*-th connected component of G
$\delta_{i}$	Size of $C_{i}$
Φ	Objective function
$\hat{V}$	Set of nodes to be dismantled
$W^{1},W^{2}$	Weights of DQN neural network
$a_{t}$	Action of DQN at *t*
$S_{t}$	Seed node selection state of DRL at time *t*
$V_{t}$	Selected nodes set to be removed at *t*
$D_{t}$	Degree vector of each node
$P_{t}$	Positive out-degree vector
$r_{t}$	Decision reward
$n_{I}$	Initial population size
$n_{b}$	Batch size in DRL algorithm
$n_{g}$	Maximum number of iterations
$n_{p}$	Population size in the EA algorithm
P	Population in evolution
K	The number of nodes to be removed
$\bar{K}$	The average connectivity of nodes
*d*	The 1st layer neurons’ number of the DQN
*l*	The 2nd layer neurons’ number of the DQN

**Table 2:** Specific experimental parameter settings for the proposed DSEDR algorithm.

Parameter	Value
Iteration number $n_{g}$	100
Evolutionary population size $n_{p}$	100
Crossover probability $p_{crossover}$	0.8
Mutation probability $p_{mutation}$	0.2
Network embedding dimension *d*	64
Training batch size $n_{b}$	512
Training discount rate γ	0.8
Training learning rate α	0.001
Importance of positive edge share λ in Φ	1

**Table 3:** Detailed information of the war network.

Network Parameter	Value
number of nodes *n*	166
number of sides *k*	1433
number of positive sides $k^{+}$	1295
number of negative side $k^{-}$	138

## Data Availability

The data presented in this study are available on request from the corresponding author due to restrictions on privacy and confidentiality, which prevent open sharing of the dataset.
